# Extracellular Vesicles and Immunity: At the Crossroads of Cell Communication

**DOI:** 10.3390/ijms25021205

**Published:** 2024-01-18

**Authors:** Noemi Aloi, Gaspare Drago, Silvia Ruggieri, Fabio Cibella, Paolo Colombo, Valeria Longo

**Affiliations:** Institute for Biomedical Research and Innovation, National Research Council of Italy (IRIB-CNR), Via Ugo La Malfa 153, 90146 Palermo, Italy; noemi.aloi@irib.cnr.it (N.A.); gaspare.drago@irib.cnr.it (G.D.); silvia.ruggieri@irib.cnr.it (S.R.); fabio.cibella@irib.cnr.it (F.C.); valeria.longo@irib.cnr.it (V.L.)

**Keywords:** extracellular vesicles, innate immunity, adaptive immunity, immunomodulation, pregnancy

## Abstract

Extracellular vesicles (EVs), comprising exosomes and microvesicles, are small membranous structures secreted by nearly all cell types. They have emerged as crucial mediators in intercellular communication, playing pivotal roles in diverse physiological and pathological processes, notably within the realm of immunity. These roles go beyond mere cellular interactions, as extracellular vesicles stand as versatile and dynamic components of immune regulation, impacting both innate and adaptive immunity. Their multifaceted involvement includes immune cell activation, antigen presentation, and immunomodulation, emphasising their significance in maintaining immune homeostasis and contributing to the pathogenesis of immune-related disorders. Extracellular vesicles participate in immunomodulation by delivering a wide array of bioactive molecules, including proteins, lipids, and nucleic acids, thereby influencing gene expression in target cells. This manuscript presents a comprehensive review that encompasses in vitro and in vivo studies aimed at elucidating the mechanisms through which EVs modulate human immunity. Understanding the intricate interplay between extracellular vesicles and immunity is imperative for unveiling novel therapeutic targets and diagnostic tools applicable to various immunological disorders, including autoimmune diseases, infectious diseases, and cancer. Furthermore, recognising the potential of EVs as versatile drug delivery vehicles holds significant promise for the future of immunotherapies.

## 1. Introduction

For a long time, extracellular vesicles have been poorly investigated, as they were considered cellular “waste”. However, with the advent of standardised purification and detection methods in the early 2000s, the field of extracellular vesicle research has experienced significant growth. These membranous nanoparticles, originating from diverse cell types, have emerged as crucial players in cell-to-cell communication, influencing cell and tissue homeostasis, responding to environmental changes, and modulating the function of target cells [1]. In recent years, EVs have been extensively studied across various physiological and pathological contexts [2]. On the basis of their cellular origin, EVs can transfer different proteins, metabolites, lipids, DNA, RNA, and miRNA between distal cells, and, indeed, they are promising as biomarkers for disease states [3]. In 1996, Raposo and co-workers were the first to describe how EVs derived from B cells could induce antigen-specific T cell responses [4]. Since then, mounting evidence has revealed the involvement of EVs in the crosstalk among immune cells and the regulation of immune responses. The immune system is a complex network comprising specific organs, different cell types, and immunomodulatory molecules. It plays a crucial role in maintaining cellular homeostasis by mediating immunological tolerance and immunomodulation, thereby enabling host defence against foreign pathogens. Mechanisms involved in the crosstalk among immune system components encompass direct cell–cell contact, the release of soluble immunomodulatory factors (cytokines and chemokines), and EV-mediated signals. These vesicles, through surface protein interactions and the transfer of encapsulated molecular cargo (including cytokines, growth factors, microRNAs, and lipids), have the capacity to modulate gene expression, molecular pathways, and the phenotype of recipient cells [5].

## 2. EV Classification and Biogenesis

**Figure 1:**
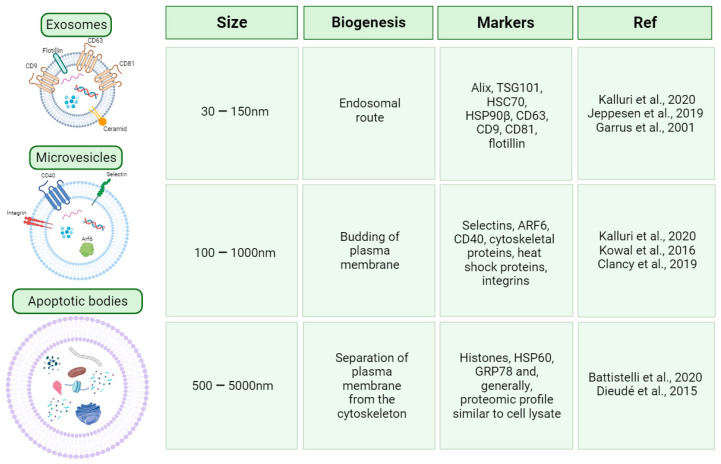
EV classification. Classification of vesicles based on size, biogenesis, and markers [8,9,10,11,12,13,14]. Created with BioRender.com.

EVs represent a heterogeneous group of particles secreted by all living cells, consisting of various classes of lipid bilayer-enclosed vesicles. These vesicles exhibit distinct biogenesis pathways and cargo enrichment [6]. The biogenesis of EVs involves three major classes, initially categorised according to their size: apoptotic bodies, microvesicles, and exosomes that differ in their cellular origin, biogenesis pathways, size ranges, and functions [7] (Figure 1).

- Exosomes: Originating from the endosomal system, they are characterised by a size of 30–150 nm and specific protein markers (e.g., CD63, CD9, and Alix) [8,9,10].
- Microvesicles: Larger in size (100–1000 nm), microvesicles are shed directly from the plasma membrane [8,11,12].
- Apoptotic Bodies: Released during programmed cell death, apoptotic bodies have a size range of 500–5000 nm and contain nuclear and cytoplasmic components [13,14].

It is worth noting that the International Society for Extracellular Vesicles issued a position statement in 2018, titled “Minimal information for studies of extracellular vesicles 2018 (MISEV2018)”, which officially recognises all these vesicles as extracellular vesicles. [15]. Overall, the biogenesis of EVs is a complex and dynamic process that varies according to the producing cell type [16]. This complex process encompasses multiple pathways and regulatory mechanisms that impact the cargo composition and functions of these vesicles in intercellular communication. Endosomes, which are vesicular structures that form from the plasma membrane, are the origin of the vesicles. Intraluminal vesicles (ILVs) derived from endosomes are subject to the control system of EV biogenesis, known as the External Sorting Complex Required for Transport (ESCRT). ESCRT consists of four multimeric complexes (ESCRT-0, -I, -II, and -III). ESCRT-0, -I, and -II sequester ubiquitinated proteins, while ESCRT-III promotes the budding of EVs [17]. The internalised vesicles merge with larger structures of the endosomal system, becoming Early Sorting Endosomes (ESEs), which undergo a maturation process leading to the formation of Late Sorting Endosomes (LSEs). Invagination of their membranes leads to the production of multivesicular bodies (MVBs). MVBs can take two distinct pathways: they may fuse with lysosomes for content degradation or merge with the plasma membrane facilitated by Ras-Associating Binding (RAB) proteins, leading to the release of vesicles contained inside. These released vesicles are referred to as small extracellular vesicles [8,18]. The biogenesis of EVs also involves the incorporation of a large number of membrane proteins on their surface during their formation in cells [19]. Among the various proteins associated with EVs, Argonaute2 (Ago2) has been identified as a key player in the biogenesis and function of EVs. Ago2 has been shown to be responsible for messenger RNA cleavage activity, stabilising miRNAs, and facilitating the packaging of secreted miRNAs into microvesicles [20,21]. Furthermore, Ago2 has been associated with extracellular vesicles and actively contributes to safeguarding miRNAs from RNase digestion in carriers such as EVs [22]. Furthermore, phospholipase D2 (PLD2) has been shown to participate in the biogenesis of EVs/exosomes across different cell types, underscoring the involvement of specific proteins in EV biogenesis [17]. Additionally, calcium has been identified as a mediator of EV biogenesis through alternate pathways in malignancy, further enhancing our comprehension of the regulatory mechanisms governing EV formation [23]. Syndecans are a family of heparan sulfate proteoglicans that interact with tetraspanin 6 to regulate the release and degradation of EVs [24]. Further investigations have revealed that the activation of EV biogenesis involves the participation of heparanase and the syndecan–syntenin–ALIX exosome pathway, influencing the selection of specific cargo for exosomes [25]. Syntenin binds with syndecan, and Alix interacts with several External Sorting Complex Required for Transport (ESCRT) proteins, particularly ESCRT-I and ESCRT-III, promoting the budding and scission of EVs [26]. The ESCRT pathway is the preferential system for the formation of vesicles. However, an ESCRT-independent pathway exists, encompassing three different processes: (i) the ceramide-dependent process, (ii) the tetraspanin-dependent process, and (iii) a pathway discovered in human embryonic kidney cells (HEK293). Ceramides have been implicated in EV biogenesis and secretion, although the precise cellular machinery orchestrating the formation of ceramide-enriched EVs remains incompletely understood [27]. Notably, the role of ceramide is cell-dependent and has not been extensively studied across all cell types [28]. The production of ceramide is under the control of the neutral sphingomyelinase (nSMase) enzyme, which hydrolyses sphingomyelin to ceramide and leads to ILV formation [28,29,30]. Another ESCRT-independent pathway is mediated by CD63 in melanoma cells and by CD82 and CD9 in HEK293 [26].

### 2.1. Proteins Involved in Immune Cells’ EV Uptake: Integrins, Immunoglobulins, and Lectins

The ability of EVs to modulate signal pathways and induce phenotypic changes in target cells has highlighted the importance of understanding the molecular mechanisms governing EV uptake into cells. Over the last few decades, researchers have extensively investigated and described several ways of EV internalisation and demonstrated that these mechanisms may depend on proteins and glycoproteins found both on vesicles and target cell membranes, whose interactions facilitate endocytosis processes such as clathrin-mediated endocytosis (CME), phagocytosis, micropinocytosis, and plasma or endosomal membrane fusion [31,32]. Recent reports have shed light on the internalisation of extracellular vesicles (EVs) by immune cells. These studies have revealed that proteins involved in cell–cell adhesion, antigen presentation, and migration, such as tetraspanins, integrins, immunoglobulins, and lectins, also regulate EV uptake in immune cell subpopulations. Morelli and colleagues demonstrated that exosomes derived by BALB/c bone marrow–dendritic cells (BMDCs) were actively internalised by murine DCs. This process is mediated by the interaction between the ICAM-1 (immunoglobulin-like protein) ligand and CD11a on exosomes and murine DC surfaces. The study has also identified the integrins CD51 and CD61 on the dendritic cell surface as regulators of exosome internalisation [33]. Other studies have demonstrated that the CD11a subunit, a component of lymphocyte function-associated antigen 1 (LFA-1)—a key regulator of critical pathways in the immune response—induces the uptake of EV in T cells following the interaction with ICAM-1 [34]. Furthermore, naive T cells internalise EVs through an interaction mechanism that involves the T Cell Receptor (TCR), CD28, and LAF-1. T cells can also incorporate DC-derived extracellular vesicles through the interactions of TCR/MHC and LFA-1/ICAM-1. These interactions also regulate the uptake of CD8^+^ T cell-derived EVs by dendritic cells [35,36,37]. The membranes of EVs are characterised by specific surface glycans, also known as glyco-signature or glycome, which depend on the EV biosynthetic process in the originating cells. The glycome plays a crucial role in EV sorting, trafficking, cell communication, cell adhesion, and immune response/evasion mechanisms [38,39,40]. It has been established that the interaction between EVs and target cells, thereby regulating their biological function, is critically influenced by the recognition of surface glycans by Glycan-Binding Proteins (GBPs). GBPs bind specific sugar epitopes on the surface, initiating signal transduction mechanisms. Two major classes of GBPs have been identified: lectins and glycosaminoglycan-binding proteins [41]. Lectins, a class of carbohydrate-binding proteins found throughout organisms from all kingdoms of life, serve various biological roles such as mediating cell–cell interactions, participating in signalling pathways, and contributing to innate immune responses against pathogens [42]. Lectins are classified into various subtypes based on their structures and the characteristic Carbohydrate-Recognition Domain (CRD). These subtypes include C-type lectins, calcium-dependent lectins, I-type lectins with a CRD similar to immunoglobulins, S-type thiol-dependent lectins (or galectins), pentraxins (pentameric lectins), and P-type lectins, which bind glycoproteins containing mannose 6-phosphate [42]. Macedo da Silva and colleagues conducted a database analysis, identifying a total of 21 human lectins on EV surfaces. These lectins belong to various families, including S-type lectins (Galectin-1, Galectin-10, Galectin-3, Galectin-4, Galectin-7, and Galectin-8), C-type lectins (CD62 antigen-like family member P, CD antigen CD162, and CD62 antigen-like family member L), collectin (Collectin-12), and some members of the F-lectin family or ficolins (ficolin-1, ficolin-2, and ficolin-3) [43]. Gerlach and Griffin have discovered that lectins play a crucial role in the recognition and uptake of EVs by recipient cells [44]. Recently, the C-type lectin DC-SIGN has been identified on monocyte-derived dendritic cells. It has been demonstrated that this lectin interacts with MUC1 expressed on breast milk-derived EV, facilitating their internalisation [45]. Similarly, another C-type lectin receptor, DEC-205, mediates the entry of EVs into dendritic cells and macrophages by recognising Galectin-5, a lectin associated with EV membranes [46]. These findings underscore the pivotal role of glycan–lectin–lectin receptor interactions in the regulation of EV uptake by immune cells.

### 2.2. The New Mediators of Immunological Response: Immune Cell-Derived Extracellular Vesicles

Extracellular vesicles derived from immune cells have emerged as key mediators of intercellular communication within the immune system. Recent findings underscored how EVs serve as a mechanism by which immune cells communicate and exert their functions, including the regulation of inflammation, antigen presentation, and immune cell activation [47,48]. A paradigmatic example has been demonstrated in the context of cancer. Extensive research has been conducted on EVs and their immunomodulatory effects, which can alter immune cell phenotypes and functions, facilitating tumour immune evasion. It is worth noting that this specific topic will not be reviewed in this manuscript, as it has been comprehensively covered in several specific recent articles [49,50,51,52]. Additionally, immune cell-derived EVs have the capability to enhance inflammation and facilitate immune cell polarisation, emphasising their pivotal role in immune regulation [53], governing both central and peripheral immune responses, and showcasing their extensive impact on immune function [48]. Different immune cells, such as macrophages, dendritic cells, T cells, B cells, NK cells, and red blood cells (RBCs), release EVs with unique compositions. These EVs have the capacity to either augment or inhibit immune responses, thereby playing a crucial role in maintaining immune homeostasis. In the upcoming paragraphs, we will offer a concise summary of the fundamental characteristics of vesicles originating from immune cells (see Table 1 for a summary).

#### 2.2.1. Monocyte- and Macrophage-Derived EVs

Monocytes originate from hematopoietic stem cells in the bone marrow and traffic via the bloodstream to peripheral tissues. There, they undergo differentiation into dendritic cells or macrophages in response to environmental challenges such as local growth factors, proinflammatory cytokines, and microbial products [98]. Several studies have been focused on the characterisation of monocyte-derived EV surface markers. In particular, it has been demonstrated that monocyte EVs carry on their surface the classical monocyte markers such as CD14, CD4, CD16, CD163, and CCR5, but lack CD63, a common EV marker [99,100]. Monocytes also serve as target cells for EVs derived from various cell types, including mesenchymal stem cells (MSCs), cardiac proliferating cells (CAPs), and quiescent endothelial cells (QECs). The cargo of these EVs, such as miRNAs, has the potential to modulate monocyte function and phenotype. Circulating monocytes have the capacity to differentiate into macrophages upon tissue recruitment. Several studies have illustrated that both monocytes and macrophages release extracellular vesicles containing shared miRNAs, such as miR-10a, miR-126, miR-27a, miR-21-5p, and miR-223, within their lumens. However, macrophage-derived EVs also display surface markers like Alix, CD63, and CD81. Furthermore, proteomic analyses conducted on EVs from both monocytes and macrophages have demonstrated the presence of alarmins. Alarmins belong to a class of chemotactic and immune-activating proteins that can bind to Toll-like receptors, galectins, annexins, and heat-shock proteins. These findings suggest a significant role for these EVs in facilitating communication between innate immune cells [54]. Macrophages exhibit the capability to internalise extracellular vesicles from a diverse range of sources, thereby inducing various activated macrophage phenotypes [101,102]. Conversely, when macrophages are exposed to chemokines, they can influence the packaging of miRNAs into newly formed EVs, subsequently regulating the function of target cells [92,103,104]. It is important to note that there is a divergence of opinions within the research community concerning the presence of microbial molecules within macrophage-derived vesicles following stimulation with microorganisms. Some authors argue that biotic stimuli lead to the incorporation of bacterial molecules into macrophage EVs, while others contend that this phenomenon may be attributed to contamination by microbial EVs during the purification process. Nevertheless, over the past decade, extensive research has been conducted on the release of extracellular vesicles by macrophages, aimed at elucidating the mechanisms underlying inflammation and immune modulation mediated by the cargo of these EVs. Indeed, macrophage-derived EVs have been shown to perform diverse biological functions, including the regulation of inflammation, immune cell activation, and the modulation of immune responses in various disease contexts [7,55,56]. Furthermore, the immunomodulatory effects of macrophage-derived EVs have been demonstrated in various conditions, such as cancer, acute kidney injury, and inflammatory disorders [57,58,59]. Macrophage-derived EVs influence macrophage activation, cytokine production, and immune cell recruitment, further emphasising their significance in immune modulation and intercellular signalling [7,60,61]. These vesicles have been implicated in chronic inflammatory diseases, such as diabetes, cancer, cardiovascular disease, pulmonary disease, and gastrointestinal disease, highlighting their immuno-modulatory effects [62]. Studies have demonstrated that exosomes derived from M1 macrophages can activate macrophage-mediated inflammation and have an impact on vascular diseases, underscoring their crucial role in immune regulation and disease pathogenesis [56,63,64]. Moreover, the cargo of EVs can influence the cellular status of recipient cells, as seen in the case of endothelial cell-derived EVs modulating the status of pericytes in response to inflammatory stimuli [65]. EVs derived from macrophages have been demonstrated to transport alarmins and modulate immune responses, with potential effects on bone homeostasis [66]. Additionally, tumour-derived EVs have been extensively studied, showing that these EVs can influence macrophage polarisation in the tumour microenvironment, affecting macrophage functional plasticity and contributing to tumour progression [105]. The alternative activation of human macrophages has been shown to enhance tissue factor expression and the production of extracellular vesicles, potentially impacting cardiovascular disease and inflammatory disorders [58,67].

#### 2.2.2. Dendritic Cell-Derived EVs

The release of EVs by antigen-presenting cells (APCs), particularly dendritic cells, is a pivotal aspect of the immune response. Dendritic cells are professional APCs known for their ability to capture, process, and present antigens to T cells, thereby initiating and modulating immune responses. The EVs released by dendritic cells have been shown to carry a cargo of bioactive molecules, including major histocompatibility complex (MHC) molecules loaded with antigens. Several studies have provided evidence for the presence of MHC molecules on EVs released by dendritic cells. For example, Zitvogel and coworkers demonstrated that EVs derived from dendritic cells express MHC class I and II molecules, as well as co-stimulatory molecules such as CD80 and CD86, which are essential for T cell activation [68]. Furthermore, it has been reported that EVs contain functional MHC-peptide complexes that can stimulate antigen-specific T cell responses [67]. These findings highlight the importance of EV-associated MHC molecules in mediating antigen presentation and T cell activation. Indeed, Théry and coworkers demonstrated that EVs derived from dendritic cells also contain a repertoire of proteins, including heat shock proteins, which can serve as chaperones for antigenic peptides and contribute to the immunostimulatory properties of EVs [69]. Robbins and Morelli investigated the cargo of EVs released by dendritic cells and demonstrated the presence of various immunomodulatory molecules such as interleukin-10 (IL-10) and transforming growth factor-beta (TGF-β), as well as regulatory proteins like programmed death-ligand 1 (PD-L1). The study highlighted the ability of these EV-associated molecules to modulate the function of immune cells, such as T cells and natural killer (NK) cells [70]. Furthermore, a study provided evidence for the presence of immunomodulatory microRNAs within EVs derived from dendritic cells. These microRNAs could regulate the expression of target genes in recipient cells, thereby exerting immunomodulatory effects on immune cell function [71].

#### 2.2.3. T Cell-Derived EVs

The role of T cell-derived EVs in immune modulation and intercellular communication has been extensively documented. These EVs, particularly exosomes, are generated from the cell surface and share characteristics with endosome-derived intraluminal vesicles (ILVs) [16]. They play a pivotal role in regulating both innate and adaptive immunity by carrying preformed mediators and nucleic acids capable of modulating the function of recipient leukocytes, parenchymal, or stromal cells [72]. Additionally, T cell-derived exosomes have been reported to inhibit effector T cell responses, suggesting their potential as therapeutic and diagnostic tools in transplantation and immune-related disorders [73]. Administration of Treg EVs via tail vein injection in mice after intra-peritoneal LPS injection demonstrated a reduction in spleen-derived myeloid pro-inflammatory IL-6 transcripts and iNOS transcripts. Furthermore, a decreasing trend in IL-1β and IFN-γ transcripts was observed, with the most significant effects seen at the higher doses administered [74]. EVs have been shown to suppress the proliferation of CD4^+^ and CD8^+^ T cells, extend allograft survival, and modify dendritic cell function, highlighting their immunomodulatory effects [75,76]. Additionally, Treg-derived EVs have been implicated in ameliorating chronic prostatitis/chronic pelvic pain syndrome in rats through immunoregulation [77]. Furthermore, Treg-derived EVs are known to carry a distinct microRNA signature capable of inhibiting CD4^+^ T cell proliferation and down-regulating relevant miR-146a-5p targets, suggesting their potential role in autoimmune diseases and transplantation [78]. Indeed, miRNAs are transferred from Tregs to DCs via Treg-derived EVs [106]. In murine CD4^+^CD25^+^Foxp3^+^ cell-derived EVs, CD25 is highly expressed, while cytotoxic T lymphocyte-associated protein 4 (CTLA-4) exhibits a lower expression level. Moreover, these EVs carry CD4^+^ T cell-specific proteins such as CD4, CD2, and MHC class I [107]. In the case of human CD4^+^CD25_high_CD127_low_ Treg cell-derived EVs, they express CD25 and the homing receptor CCR4, with reduced levels of CD4 and CTLA-4, and they do not contain Fas-ligand [108]. CTLA-4, an immune checkpoint protein highly expressed on Treg cell membranes, plays a crucial role in the Treg-mediated suppression mechanism [109]. A study by Tian and coworkers investigated the cargo content of EVs released by regulatory T cells (Tregs) and identified the presence of immunomodulatory molecules, including the cytokine interleukin-35 (IL-35), which is known for its suppressive effects on effector T cell function. Furthermore, the study demonstrated that EV-associated IL-35 could mediate immunosuppressive effects on target immune cells [79]. In the context of tumour therapy, T cell-derived EVs are able to trigger a local immune response in the tumour microenvironment, leading to tumour regression [80].

#### 2.2.4. B Cell-Derived EVs

B cell-derived EVs have been implicated in various physiological and pathological processes, including immune responses, cancer progression, and regenerative medicine. Mass spectrometry analysis has revealed several components derived from B cells, including MHC-I, MHC-II, CD20, CD45, B Cell Receptor (BCR), and proteins of the complement system [79]. Furthermore, under specific conditions, B cells release a greater quantity of extracellular vesicles [110]. These attributes underscore the critical role of B cell-derived EVs as essential messengers in the adaptive immune response [111]. These EVs, which carry MHC-II molecules, engage in interactions not only with nearby T lymphocytes but also with distant antigen-presenting cells (APCs) by means of the connection between exosomal BCR and APC Fc receptors [81]. Furthermore, these EVs contain miRNAs (as miR-223, miR-155, and miR-202-3p) and other regulatory RNA species that can influence the gene expression of recipient cells, thereby modulating their function [82]. This regulatory role extends to T cells, where B cell-derived EVs can impact T cell differentiation and immunomodulatory activity [83]. It has also been observed that EVs released by B cells activate DCs because they carry MHC-II, triggering cascade activation of T CD4^+^ cells and NK cells, with consequent activation of CD8^+^ T lymphocytes, which exercise their killing response [81,84,85]. Additionally, these EVs have been shown to selectively impair lymphocyte responses to interleukin-2 [86] and to affect mRNA expression and function of B-lymphocytes, exerting differential expression of relevant genes and modulating cell function [87]. Furthermore, B cell-derived EVs have been found to play a role in antigen-specific immune suppression, mediated by T CD8^+^ cell-derived exosomes, highlighting their involvement in immune tolerance and immunosuppression [88]. Phan and coworkers demonstrated that stimulation of B cells with CD24 and BCR increased EV production, sensitising the neighbouring B-lymphocytes with their subsequent activation in the antigen interaction [112]. These EVs are also vehicles of a monomeric form of IgM antibodies different from the known pentameric one, which can open new perspectives in immune-related activities [113]. Furthermore, in vivo experiments in mice have revealed that EVs isolated from cultured primary B cells can be captured from macrophages of the spleen binding CD169, opening new scenarios in the immune response to exosomal antigen [114].

#### 2.2.5. Natural Killer (NK) Cell-Derived EVs

Natural killer (NK) cells are immune cells that play a key role in immune surveillance and host defence against tumours and pathogens. The NKs release bioactive molecules with cytotoxic activity, whose function is to destroy target cells. NK-derived EVs (NK-EVs) have been isolated from the NK92 cell line and peripheral blood mononuclear cells (PBMCs), and it has been demonstrated that they contain NK markers and lytic enzymes such as Perforin (PFN), Granzyme A (GZM-A), Granzyme B (GZM-B), and Granulysin (GNLY) [115,116,117,118,119]. In the last decade, research findings have clarified the roles of NK-EV lytic enzymes. In particular, PFN associated with NK-EV inserts itself into the membrane of target cells, generating pore formation and releasing GZM-B in the cytoplasm; both enzymes may activate caspase-dependent and independent apoptosis pathways in target cells. On the other hand, GZM-A activates apoptosis pathways inducing ROS release and DNA damage. GNLY can either bind the membrane of the target cell or enter through the polyperforin pores. This enzyme induces alterations in the membrane potential of host cells by damaging the endoplasmic reticulum and compromising mitochondrial activity, resulting in the activation of the caspase 7-induced apoptotic pathway. Furthermore, NK-EV surface ligands, such as CD95 or Fas L, are able to interact with target cell receptors. Ligand-receptor binding activates the apoptotic pathways by the Death Inducing Signal Complex (DISC). The DISC complex induces two signalling pathways: the first one mediated by caspase-9, -3, and -7, and the second by apoptosome formation, followed by caspase-3 and -7 effector activity. All these findings suggest that NK-EVs constitute a further pathway for apoptosis activation in target cells [89]. It is important to emphasise that accumulating data has revealed the role of NK-EVs as novel contributors to cancer immunotherapy, with a growing body of evidence indicating their participation in the antitumour activity of NK cells [88]. The activation of NK cells hinges on a delicate balance between activating and inhibitory signals generated by the engagement of distinct receptors. NKG2D is a C-type lectin-like activating receptor expressed on NK cells, γδ T cells, and CD8^+^T cells and represents a major recognition receptor for the detection and elimination of virus-infected and cancer cells. The expression of NKG2D ligands (NKG2DLs) serves as a “danger signal”, marking cells for immune cell attack [120]. Tumour-derived extracellular vesicles have been found to activate or inhibit NK cell functions, depending on their cargo or surface molecules, thereby influencing the tumour microenvironment [121]. Moreover, NK-EVs have been shown to use multiple cytotoxic proteins and killing mechanisms to target cancer cells, indicating their potential as therapeutic agents in cancer treatment [122]. These vesicles have also been implicated in immune surveillance, as NK cells dressed with vesicle-associated MICA (MICA is the most polymorphic NKG2DL) become susceptible to autologous NK cell lysis [90]. Additionally, NK EVs have been shown to reproduce key functions of their parent NK cells [123] and to contain Natural Killer Cell Granule Protein 7 (NKG7), a gene critical for controlling cancer initiation, growth, and metastasis. NKG7 function in natural killer was linked with its ability to regulate the translocation of CD107a to the cell surface and kill cellular targets [91].

#### 2.2.6. Red Blood Cell-Derived EVs

While red blood cell (RBC)-derived extracellular vesicles were initially thought to be remnants of the maturation process, recent research suggests a more dynamic role. They are formed in cytoskeleton-free regions of the RBC membrane and are suggested to be a lipid raft-based process induced by ATP loss, a process known as eryptosis [91]. The biogenesis and characterisation of stored RBC-derived EVs have been a subject of great interest, particularly in the context of blood transfusion and its potential impact on immunomodulation in patients with cancer [124]. Furthermore, RBC-derived EVs have been associated with coagulation activation pathways, influencing immune and coagulation parameters in critically ill transfused patients [93,94] and their impact on B lymphocyte survival and plasma cell differentiation [95]. Additionally, RBC-derived EVs have been found to induce human mast cell activation and the production of multiple inflammatory mediators, indicating their potential role in immune response modulation [96]. RBC-derived EVs also promote the pro-inflammatory polarisation of macrophages with the release of inflammatory cytokines Tumour Necrosis Factor-α (TNF-α), interleukin-6 (IL-6) and interleukin-1β (IL-1β) through the TLR4–MyD88–NFκB–MAPK pathway [92].

Moreover, these EVs have been implicated in atherosclerosis, increasing mitogen-driven T cell proliferation in peripheral blood mononuclear cell cultures [97]. Furthermore, RBC-derived EVs have been explored for their therapeutic applications, with a focus on their potential role in the treatment of various diseases, including atherosclerosis and cerebral ischemia [125]. The formation of RBC-derived EVs has been associated with oxidative stress and an increase in intracellular calcium concentration, providing insights into the mechanisms underlying their production [126]. The potential of RBC-derived EVs as theranostic tools in kidney disease has also been explored, emphasising their multifaceted roles in disease pathogenesis and diagnosis [127]. Furthermore, the biology of RBC-derived EVs has been a subject of relevant interest, with a special focus on their potential application in treatment [128]. In fact, different studies propose that these EVs carry a cargo of biologically active molecules, including miRNAs, proteins, and lipids. In this context, RBC-derived EVs demonstrate remarkable safety, making them reliable clinical carriers due to their excellent biocompatibility. Loaded with RNA molecules, they exhibit prolonged stability and maintain their functional capacity over extended durations. Furthermore, RBC-derived EVs hold significant promise for drug delivery platforms due to their ability to penetrate anatomical barriers and display substantial budding [129].

### 2.3. The Impact of Pollution on Extracellular Vesicle (EV) Biogenesis and Function

Extracellular vesicles play a crucial role in mediating the impact of pollutants on biological systems. The cargo of EVs, including microRNAs and proteins, has been implicated in modulating tissue crosstalk, stress-induced diseases, and cellular interactions, underscoring the potential of EVs as mediators of pollutant-induced effects [130,131,132]. Recently, research on the impact of polybrominated diphenyl ethers (PBDEs), a widespread environmental pollutant, on EVs has been extensively investigated. For instance, BDE-47 has been found to modulate small EV (sEV) biogenesis and their miRNA cargo, exacerbating the lipopolysaccharide (LPS)-induced pro-inflammatory response in THP-1 macrophages [133,134]. Purified sEVs from BDE-47-treated THP-1 macrophages may affect the expression of surface markers in a naïve resting M(0) macrophage cell line, impairing the ability to make immunological synapses and present antigens downregulating the expression of HLA-DR and CD209 antigens [133].

### 2.4. Extracellular Vesicles (EVs) in Pregnancy

During pregnancy, immunological changes occur to support the adoption of the maternal immune system by the semi-allogeneic foetus within the pregnant uterus. Placental development is fundamental to establishing and maintaining close contact between foetal and maternal cells at the maternal–foetal interface, allowing well-regulated immune interactions between the pregnant woman and the foetus [135]. At the same time, the placenta serves as an immune barrier, providing protection against infections [136]. These mechanisms require a tightly controlled transport of biological information and molecular signalling between the mother, foetus, and placenta to ensure a favourable immunological environment [137]. Among the complex molecular networks that are established during pregnancy, extracellular vesicles are considered key elements of intercellular communications. Their molecular cargo regulates the crosstalk between the embryo and the uterine wall by initiating the processes of angiogenesis and tissue remodelling essential for early pregnancy [138]. Accumulating evidence shows a crucial traffic of extracellular vesicles (EVs) between the feto-placental unit and maternal immune cells in both regional and distal locations. This suggests the existence of a distinctive communication system based on EVs, which actively contributes to the modulation of maternal immune responses during pregnancy. Considering the central role of the placenta during pregnancy, it is essential to highlight its contribution to the generation of extracellular vesicles, such as placenta-derived extracellular vesicles (P-EVs). P-EVs are produced during the entire course of pregnancy and can be detected in maternal circulation as early as approximately 6 weeks of gestational age [139]. The P-EVs can be isolated from the blood of pregnant women by means of the presence of a specific surface protein named placental-alkaline phosphatase-positive (PLAP) [140]. P-EVs have been found to continuously increase in maternal circulation over the first trimester of pregnancy, indicating their potential as biomarkers for monitoring placental health during early pregnancy [140]. PLAP+-EVs contain immunomodulatory proteins and microRNAs that orchestrate a delicate balance between pro-inflammatory and anti-inflammatory responses, essential for protecting the foetus while allowing necessary maternal immune responses [141]. These effects are promoted through the transfer of regulatory molecular signals to immune cells, including regulatory T cells (T-regs), dendritic cells, and macrophages, which are influenced by P-EVs to shape the immune uterine environment [142]. At the same time, in vitro studies have shown that placental cells can be a target of immunomodulatory signals carried by EVs that originate from foetal tissues [143]. In this study, Yadava and collaborators have speculated that this mechanism may be implicated in the induction of delivery by foetal signals through the activation of the pro-inflammatory pathway in the placenta [143]. P-EVs have received special attention, mainly due to their reported roles in both normal pregnancies and pregnancy-related disorders [144]. Recent studies have highlighted the potential utility of P-EVs in the diagnosis and monitoring of pregnancy-related complications, including gestational diabetes mellitus [145] and pre-eclampsia [146]. Furthermore, EVs have been associated with the prediction of preterm birth, highlighting their role in foetal development [147]. The role of P-EVs and their potential use as biomarkers is an area of growing interest in the field of medicine. However, their biomarker potential could also be exploited in the context of environmental exposure assessments during pregnancy and their health-related consequences [148]. In particular, their ability to transport microRNA from the maternal to the foetal side could be the basis of epigenetic alterations that are the basis of variations in health trajectories. In relation to the recent expansion surrounding EV discoveries, emerging evidence supports the utility of evaluating EVs as exposure biomarkers in humans. As described in recent studies, potential EV molecular biomarkers have been identified in response to cigarette smoking in chronic lung diseases and to exposure to particulate matter [149,150]. In the above-described context, further investigations are needed to increase knowledge on the potential role of P-EVs in developmental immunotoxicity.

### 2.5. Host–Pathogen EV-Mediated Interaction

Extensive research has been conducted on the modulatory effects of pathogen-derived extracellular vesicles on the host immune response. Host–pathogen interactions involve intricate processes wherein viruses, bacteria, fungi, or parasites engage, adapt, and sustain themselves within host organisms. This dynamic phenomenon is influenced by a diverse array of factors that ultimately determine the course of infection, ranging from complete pathogen elimination (resulting in a cure) to the development of pathologies and, in severe cases, the demise of the host. Several studies have demonstrated the export of virulence factors via EVs across a wide spectrum of pathogens. For instance, the fungus Cryptococcus neoformans releases glucuronoxylomannan (GXM) within EVs, a capsule component with immunomodulatory properties [151]. Pseudomonas aeruginosa employs outer membrane-derived vesicles (OMVs) to transport virulence factors, including hemolytic phospholipase C, β-lactamase, and alkaline phosphatase, directly into the host cytoplasm by fusing with lipid rafts in the host plasma membrane. EVs from Trypanosoma cruzi contain essential factors for parasite survival, such as trans-sialidases (TS), mucin, mucin-associated surface protein (MASP), cruzipain, and phosphatases [152,153]. EVs from ring-stage Plasmodium falciparum-infected red blood cells carry PfEMP1—an important factor for erythrocyte adherence—and induce transcriptomic changes in recipient monocytes [154]. Moreover, research by Toda and coworkers revealed that splenic fibroblasts exhibited higher binding of P. vivax-infected erythrocytes when exposed to plasma-derived EVs from infected patients compared to EVs from healthy individuals [155]. Toxins can also be disseminated through EVs, as demonstrated in microorganisms such as Bacillus anthracis (anthrax toxin) [156], Staphylococcus aureus (staphylococcal alpha-toxin) [157], and Listeria monocytogenes (pore-forming toxin listeriolysin O) [158], potentially exhibiting greater cytotoxicity than the purified toxin alone [159]. Additionally, host EVs derived from Salmonella-infected cells stimulate pathogen-specific Th1-type responses in vivo, indicating their role in the immune defence against pathogens. Moreover, human neutrophils produce antifungal EVs against Aspergillus fumigatus, suggesting a role for EVs in the defence against fungal pathogens. Furthermore, pathogen-derived EVs have been shown to counteract the attack of the complement system [160] and regulate the responses of crucial cells, including macrophages and dendritic cells [161,162,163,164].

### 2.6. EVs as Drug Delivery System

The clinical potential of extracellular vesicles as drug delivery systems is a topic of growing interest in biomedical research, and their possible therapeutic applications across a range of medical conditions have been suggested [165]. In recent years, the new knowledge on the endogenous properties of extracellular vesicles, such as biocompatibility, stability, and the ability to circulate through the bloodstream and cross both the blood–brain and blood–tumour barriers, has made them attractive candidates for the development of innovative therapeutic approaches in medicine. Therefore, different strategies based on chemical–physical processes or bioengineering of cargo and surface membranes were carried out in order to develop innovative therapeutic approaches, especially for the treatment of immuno-inflammatory pathologies. The main strategies allowing the production of a high quantity of engineered EVs in immunotherapies are as follows: (i) the in vitro manipulation of parental cells by genetic engineering technologies or preconditioning processes with specific signal molecules, which stimulate the secretion of modified EV; (ii) the EV’s membrane surface modification by either chemical surface engineering or the new membrane tethering technologies (MTFP); and (iii) the EV cargo alteration by introducing exogenous molecules such as chemotherapeutic drugs, bioactive peptides, mRNAs, miRNAs, etc., into the vesicle’s lumen. This targeted approach could reduce unwanted side effects associated with conventional therapies and enhance treatment efficacy. Furthermore, EVs can lean on their biological camouflage to evade the immune system and reach their target without being recognised as foreign bodies. This camouflage ability, combined with their biocompatible nature, makes EVs a potentially safe and efficient drug delivery system [166].

EVs as carriers or therapeutic agents can be applied in different fields of medicine, from autoimmune diseases to cancer. Immune cell-derived extracellular vesicles encompass various subtypes, including dendritic cell-derived EVs, blood cell-derived EVs, and macrophage-derived EVs. These vesicles carry crucial components such as MHC I and II for antigen presentation, along with necessary co-stimulatory molecules [167,168]. Adhesion molecules like CD11b, CD9, and lactadherin, which guide EVs toward effector cells, are also released through selective enrichment by immune cells [169]. EVs can mediate the transfer of molecules that influence tissue remodelling, attenuating damage through their capacity for drug delivery. Researchers have explored the modulation of immune cells by extracellular vesicles derived from mesenchymal stem cells (MSCs) and their clinical potential in the management of inflammatory diseases [170,171,172]. Notably, macrophage-derived EVs, being integral to the immune system, exhibit superiority over other microparticles in terms of drug delivery as they evade phagocytic elimination. Conjugated with curcumin and albumin, they demonstrated the ability to normalise inflammatory markers in a skin inflammation model that mimics psoriasis [173]. Studies on rheumatoid arthritis have demonstrated that, on the one hand, bone marrow-derived DC EVs exert an anti-inflammatory effect, recovering the arthritis through the MHC II complex; on the other hand, by combining the antibody anti-reactive oxygen species collagen type II with neutrophil-derived EVs, it can be possible to access the cartilage [174]. A potential natural drug for the treatment of Lupus Erythematosus (LE) is curcumin; in fact, B cell lymphoma-derived EVs, encapsulated with curcumin, exert anti-inflammatory activity in an LPS mouse model, paving the way for the use of this compound for the treatment of glomerulonephritis by LE [175,176]. Furthermore, extracellular vesicles have surfaced as promising candidates for clinical therapeutics in the realm of bone regeneration. Studies have demonstrated their capacity to regulate both innate and adaptive immunity, as well as foster immune tolerance in immune-competent animals [177].

A large set of data has been recently produced in the field of the therapeutic application of EVs from innate and adaptive immune cells in the field of cancer immunotherapy. Jung and colleagues have recently reviewed the current advances in this field that will not be addressed in this review. In this interesting article, the authors systematically described the different cancer immunotherapeutic platforms developed in recent years, collecting data related to the different immuno-derived EV subtypes, the engineering technology performed, and the therapeutic effects obtained on different tumour types [178].

## 3. Conclusions

In summary, both in vivo and in vitro studies on extracellular vesicles have significantly advanced our comprehension of these tiny, membrane-encased particles and their roles in intercellular communication and various physiological processes. These investigations have illuminated the diverse functions of EVs in health and disease, encompassing their participation in immune responses, tissue regeneration, and the progression of cancer. It is noteworthy that EVs have been shown to play a multifaceted and pivotal role in immunity, acting as messengers that convey vital information among immune cells. They fine-tune immune responses, contribute to immune regulation, and maintain homeostasis. Their ability to transport a diverse array of proteins, nucleic acids, and other bioactive molecules underscores their significance in orchestrating intricate and tightly regulated immune processes. In vitro studies have provided crucial mechanistic insights into EV biogenesis, cargo loading, and release mechanisms, offering prospects for the development of potential diagnostic and therapeutic applications, such as EV-based biomarkers and drug delivery systems. Although we have extensively described the advantages and therapeutic potential of extracellular vesicles from immune cells, the possibility of achieving broad clinical applications requires (i) a more profound comprehension of the molecular mechanisms mediated by natural immune EVs, (ii) the implementation of a multidisciplinary approach to enhance EV engineering strategies, and (iii) the establishment of standardised production processes compliant with “Good Manufacturing Practice” guidelines to assure safety conditions. Ongoing research into EV biology holds immense potential for unveiling innovative therapeutic strategies and deepening our understanding of immune-related disorders, which will advance the fields of immunology and healthcare.

## Figures and Tables

**Table 1:** Effects of immune cell-derived EVs on target cells.

Cell Type	Effect of Cell Type-Derived EVs on Target Cells	Reference
Monocytes and Macrophages	- Involvement in the innate immune cell’s communication	[54]
	- Regulation of inflammation, immune cell activation, and modulation of immune responses in various disease contexts	[55,56]
	- Immunomodulatory effects in cancer, acute kidney injury, and inflammatory disorders	[57,58,59]
	- M1-derived EVs induce macrophage activation, cytokine production, and immune cell recruitment	[60,61]
	- Implication in chronic inflammatory diseases (diabetes, cancer, cardiovascular disease, pulmonary disease, and gastrointestinal disease)	[62]
	- Activation of macrophage-mediated inflammation and effects on vascular diseases	[63,64]
	- Modulation the status of pericytes in response to inflammatory stimuli	[65]
	- Regulation of bone homeostasis	[66]
Dendritic Cell	- T cell stimulation and antigen-specific T cell responses	[67,68,69]
	- Modulation of T cells and NK cell function	[70]
	- Immunomodulatory activity mediated by miRNA cargo on immune target cells	[71]
T Cell	- Modulation of leukocytes, parenchymal, or stromal cells functions	[72]
	- Inhibition of effector T cell responses	[73]
	- Reduction of IL-6, iNOS, IL-1β, and IFN- γ transcripts in spleen-derived myeloid cells	[74]
	- Suppression of CD^4+^ and CD8^+^ T cell proliferation	[75]
	- Regulation of DCs function, highlighting their immunomodulatory effects	[76]
	- Treg-derived EVs ameliorate chronic prostatitis/chronic pelvic pain syndrome in rats	[77]
	- Involvement in modulation of autoimmune diseases and transplantation by inhibition of CD4^+^ T cell proliferation and relevant miR-146a-5p targets	[78]
	- Immunosuppressive effects of Treg EVs on target immune cells	[79]
	- Induction of tumour regression in tumour microenvironment	[80]
B cell	- T cell interaction and immunomodulatory activity in T cell differentiation	[81,82,83]
	- Activation of DCs, T CD4^+^ and NK to induce TCD8^+^ killing response	[83,84,85]
	- Inhibition of lymphocyte response to interleukin-2	[86]
	- Modulation of gene expression in B-lymphocytes	[87]
Natural Killer	- Involvement in immune tolerance and immunosuppression	[88]
	- Antitumoral activity as effectors of NK cells	[88]
	- Activation of caspase-dependent or independent apoptosis pathways	[89]
	- Implication in immune surveillance	[90]
	- Regulation of cancer initiation, growth and metastasis as well as NK cells	[91]
Red Blood Cell (RBC)	- Macrophage pro-inflammatory polarisation	[92]
	- Activation of coagulation pathways	[93,94]
	- Impact on B lymphocyte survival and plasma cell differentiation	[95]
	- Human mast cell activation and induction of inflammatory mediators	[96]
	- T cell proliferation in peripheral blood mononuclear cell cultures	[97]
